# Toxicity and Femininity in Love Island: How Reality Dating Shows Perpetuate Sexist Attitudes Towards Women

**DOI:** 10.3389/fsoc.2021.641216

**Published:** 2021-06-02

**Authors:** Alicia Denby

**Affiliations:** The University of Manchester, Manchester, United Kingdom

**Keywords:** love island, intimate relationships, reality TV, toxic relationships, emotions, heteronormativity

## Abstract

Using episodes of ITV2’s *Love Island* (2016–2020) as a case study, this paper explores the extent to which reality dating shows perpetuate sexist attitudes towards women through a heteronormative focus. Examining the operation of gender roles in *Love Island*, in the context of emotional intimacy and physical intimacy, this paper proposes that the performance and portrayal of heteronormative ideals disadvantage women. Specifically, by presenting female contestants as overly emotional and irrational, outdated stereotypes surrounding emotionality and hysteria are reproduced within *Love Island*. Moreover, the stigmatization of sex-positive women in *Love Island* demonstrates the existence of a sexual double standard wherein male contestants are celebrated for their sexual prowess, while female contestants are shamed and deemed unruly, by virtue of their sexual dominance. Fundamentally, this paper contributes to research on contemporary sexualities by demonstrating how, despite the cultural shift towards greater gender equality, traditional gendered ideals continue to exist in heterosexual relationships, which serve to disadvantage women.

## Introduction

In sociological research and popular culture, “masculinity” and “femininity” are conceptualized to be reflexive constructions, adapting alongside social, cultural, and political developments (Kaplan et al., 2017). In recognition of such developments, sociological interest in the field of intimacy addresses the extent to which intimate relationships alter under late-modern conditions, characterized by virtues of freedom and autonomy (Giddens, 1992; Beck and Beck-Gernsheim, 1995; Bauman, 2003). Indeed, while scholarship suggests intimate relationships have become detraditionalized in Western societies, departing from traditional heteronormative ideals, toward greater autonomy and diverse sexualities (Giddens, 1992), this paper draws attention to the existence of traditional and heteronormative ideals in contemporary intimacy, thus supporting suggestions that “romantic relationships are the arena in which traditional gender ideologies are upheld most strongly” (Sweet, 2019: 855) and are, therefore, “the place to look for the ongoing animation of traditional ideologies” (ibid.,).

According to traditional gendered scripts, intimate relationships were divided into masculine and feminine variables, whereby men performed an instrumental and dominant role, while women adopted emotionally expressive qualities (Parsons, 1959; Haywood, 1998). However, as gender roles are not universally fixed but rather “intricately connected to social, cultural and economic contexts” (Haywood, 2018: 26), with cultural trends toward greater gender equality, roles within intimate relationships became diversified from the heteronormative ideal, marking the transition towards greater autonomy and diverse sexual identities (Giddens, 1992). Yet, despite an apparent transition towards greater flexibility in gender roles, traditional and heteronormative expectations continue to dominate heterosexual intimate relationships. As such, while men are encouraged to express their emotions and sensitivity in contemporary relationships, there is a simultaneous expectation for men to “man up,” in fear of showing weakness and vulnerability (Gough, 2018: 40). Similarly, while women are sexually liberated and able to “make the first move,” an expectation remains that men should initiate dating, akin to the expectation that men should confirm the relationship when asking to become “exclusive” and proposing marriage (Glenn and Marquadt, 2001). Hence, with greater ambiguity in how gender roles operate within contemporary intimate relationships (Glenn and Marquadt, 2001), this paper uses episodes of *Love Island* as a case study to examine the operation of gender roles in contemporary intimate relationships, conceding that the representation of gender roles in *Love Island* perpetuate heteronormative ideals that serve to disadvantage women.

## Research Question and Research Design

### R1) To What Extent Does the Representation of Gender in *Love Island* Perpetuate Sexist Attitudes Towards Women?

To address this question, episodes of ITV’s *Love Island* were analyzed to uncover patterns and trends in contestants’ attitudes and behavior. The premise of *Love Island* surrounds observing a group of young, heterosexual singles, referred to as “islanders,” isolated in a luxurious villa for 8 weeks, in the pursuit to find love. Islanders “couple up” with a contestant of the opposite sex during the initial coupling ceremony, and over the duration of the series, couples are put to the test by new arrivals, eliminations and weekly “re-coupling” ceremonies. Finally, the series draws to an end when the public vote for a couple to be chosen as the winners of *Love Island,* and the winning couple is put to one final test when presented with the decision to either choose the £50,000 prize money or choose love. Attracting criticism across its’ six series’, episodes from series 2 to series 6 of *Love Island* were selected as the sample, with scenes that caused conflict and controversy chosen for textual analysis. Due to practical limitations, it would not be feasible for all episodes to undergo textual analysis, therefore, following the recommendation for qualitative samples to “be of a size that can be managed in practical terms” (Mason, 2002, cited in Emmel, 2013: 140), ten scenes were decided based on the media coverage attracted. Upon selection, scenes were transcribed verbatim and conversations were textually analyzed to identify patterns in how gender roles are presented and discussed in *Love Island.*

Although reality television purports to present real life, this paper considers how reality TV exists as a “mediated reality” (Cato and Carpentier, 2010) wherein television offers a reflection of real life, albeit in a semi-artificial context where scenes are edited, exaggerated and manipulated for entertainment purposes. In acknowledgment of this, the following discussion accepts that while reality television is not entirely authentic, contestants display real life attitudes and emotions, thus *Love Island* proves a valuable source to capture societal attitudes. However, given the subjective nature of qualitative analysis, methodological issues should be acknowledged in anticipation of the preceding discussion, particularly regarding how the analysis is based on a sole interpretation, constructed “through the lens of [ones] own attitudes values, belief, biases, heuristics, and stereotypes” (Morgan and Dennehy, 2004: 375, cited in Cato and Carpentier, 2010). Hence, as data is analyzed based on subjective interpretation, the following analysis does not represent an objective truth nor the attitudes of a wider population.

## Analysis and Discussion

### “You’re Losing Your Head”: Gaslighting the “Crazy” Woman

Considering the criticism surrounding reality dating shows, with a specific focus into how reality television shows often present stereotypical gender roles (Anderson and Ferris, 2016) a particular concern upon analysis of *Love Island* surrounds how the gendered stereotype of the “crazy” woman is perpetuated and normalized within the villa, especially as this stereotype is frequently used against women in abusive relationships (Sweet, 2019). Certainly, the stereotype of the “crazy” woman has historical roots in societal institutions, wherein women were perceived to be overly emotional, hysterical and unstable (ibid.,). Historically, women were cast as witches and diagnosed “insane” by virtue of their disobedience to feminine ideals and rebellion “against the female role” (Showalter, 1981: 324). As such, cultural ideals of “madness” were fundamentally shaped by patriarchal structures (Zaccour, 2018: 59), as a means to reassert men’s power and control over women and prevent non-conformity to the feminine ideal. Indeed, while the stereotype of the “crazy” woman has evolved, consistent with social and cultural developments towards gender equality, despite such changes, this stereotype continues to draw upon deviations of femininity, particularly surrounding emotional instability and irrationality. In the field of medicine, women’s health concerns are frequently dismissed as irrational and pathologized to be psychosomatic (Kempner, 2014; Sweet, 2019), demonstrating the ways in which women are perceived as overly emotional, irrational and dramatic. Moreover, in the context of law, emotional instability is used as a defense against women, especially in cases of domestic abuse (Sweet, 2019), and custody proceedings (Zaccour, 2018). Fundamentally, women who deviate from a submissive ideal of femininity are labeled in this way and, as demonstrated by events in *Love Island*, this stereotype persists to the present day, where emotional women are perceived to be crazy and irrational.

With existing stereotypes surrounding women’s emotional instability, jealousy, and paranoia, men are able to call on women’s emotions and label them “crazy” when gaslighting them (Sweet, 2019). Defined as a form of emotional abuse where a victim is manipulated to the extent that they begin to “question their thoughts, memories and events occurring around them” (York-Morris, 2017), gaslighting is increasingly common in intimate relationships and, as reality television reflects real-life attitudes and behaviors, gaslighting is a common trope in *Love Island,* especially concerning how women are led to believe they are paranoid or “crazy” when suspicious of their partners’ infidelity and deceit.

Consistent with this behavior, male islanders in *Love Island* often deny their partners’ suspicions of infidelity and accuse their partner of being paranoid or irrational, despite their suspicions being justified. During series five of *Love Island*, Jordan Hames accused Anna Vakili of overreacting when Anna confronted Jordan upon discovering that he had attempted to seek a romantic connection with fellow contestant, India Reynolds, only two days after asking Anna to be his girlfriend. Despite previous scenes showing Jordan admitting his attraction towards India to fellow islander, Curtis Pritchard, Jordan denied this to Anna and when questioned on the nature of his conversation with India, Jordan trivialized Anna’s suspicions by asking “am I not allowed to have a conversation with someone? (Series 5 Episode 44, 2019: 43 min 59). In doing so, Jordan led Anna to believe she was paranoid, and her suspicions irrational. Further, although Jordan eventually admitted to Anna that he was interested in India, Jordan later denied this when asking “when did I say that?” (Series 5 Episode 44, 2019: 44 min 11), thus encouraging Anna to question her recollection of the event.

Anna: You like her? Maura just told me.

Jordan: I’m having a conversation with her, I never said that I like her.

Anna: You just asked me out, and you like her? Is that how much of a f***ing idiot you are?

Jordan: I’m having a conversation.

Anna: What’re you talking about then? I have the right to know, you’re my boyfriend.

Jordan: I’m just talking about how I feel, which I tried to explain to you today but you started losing your head about it.

Anna: You ask a girl out, you ask a girl to be your girlfriend and 2 days later you’re hitting on her? You tried to tell me today that you like another girl? 2 days, 2 days! What kind of guy asks a girl to be their girlfriend then 2 days later starts cracking on with someone else?

Jordan: Get out of my face. I’m not cracking on with her, I’m just having a conversation. Am I not allowed to have a conversation with someone?

Anna: You have a f***ing girlfriend, you idiot.

Jordan: I’ve never said that I like the girl. When did I say that I like her?

Anna: You just said to me there that you were trying to tell me today.

Jordan: When did I say that? What did I say? You’re the most negative person I’ve ever met in my whole life. Why’re you shouting about like that?

Anna: Why am I shouting about? Maura has come up to me and said that Curtis told her that you liked India and then I come up to you and you’re all like I was gonna tell you today. You’ve been avoiding me for 2 days, you’ve made me feel like a piece of s**t, you’re an embarrassment.

Jordan: Maybe if you weren’t so negative. All you are is negative all the time and it’s so boring.

(Series 5 Episode 44, 2019: 42 min 30–45 min 00)

Similarly, during series four, *Love Island* received criticism following an incident between Adam Collard and Rosie Williams, in which the domestic abuse charity Women’s Aid accused Adam of displaying “warning signs” of gaslighting (McGrath, 2018). Prior to this incident, Adam and Rosie had been “coupled up,” but upon the arrival of new islander Zara McDermott, Adam distanced himself from Rosie, to instead pursue his interest in Zara. Further fueled by Zara choosing Adam to accompany her on a date, Rosie became suspicious of Adam’s relationship with Zara and, noticing a change in Adam’s behavior, Rosie confronted Adam to vocalize her concerns. In response, Adam trivialized Rosie’s feelings and perceived her to be insecure when telling Rosie “I don’t need to reassure you” (Series 4 Episode 14, 2018: 12 min 05) and accusing her of “looking into everything” (Series 4 Episode 14, 2018: 13 min 38), despite Rosie’s suspicions being justified.

Rosie: What’s actually going on with you?

Adam: What do you mean?

Rosie: You literally haven’t spoken to me all day.

Adam: How’ve I not spoken to you all day?

Rosie: You’ve just ignored me basically.

Adam: I haven’t ignored you. I don’t need to reassure you.

Rosie: I’m not asking you to reassure me, but it would be nice for you to speak to me like normal, like you haven’t acted normal for some reason.

Adam: I’ve been exactly myself today. I’ve been exactly how I normally am.

Rosie: *sighs* Okay.

Adam: Why’re you on the defence?

Rosie: I’m not, I’m just like.

Adam: If you’re telling me you haven’t had a bee in your bonnet when I came over there. It’s ridiculous.

Rosie: What do you mean bee in my bonnet?

Adam: You’re just being arsy from the start.

Rosie: I’m not being arsy.

Adam: You’re sitting over there with a face like a smacked a**e. You can tell a mile off.

Rosie: I was laughing and smiling with Dani.

Adam: Oh, you weren’t. I’m not daft, I know when there’s something up.

Rosie: Yeah there is something up, because you haven’t spoken to me all day. You’ve been different and you can’t say you haven’t been different.

Adam: I haven’t been different.

Rosie: Oh my God Adam you definitely have, and everyone can see it.

Adam: As soon as I came over there, that was arsy from the minute.

Rosie: Because you literally sat far away from me as if you didn’t want to speak to me.

Adam: No, I didn’t, as soon as I spoke about the date, it was a good date.

Rosie: I’m glad you had a good date, it’s not a thing for me.

Adam: It is.

Rosie: It’s genuinely not a thing.

Rosie: You want to know something, you said you came in here for something real, yeah?

Adam: Yeah.

Rosie: Yeah, you’re not going to get something real if you’re always looking for more.

Adam: *rolls eyes*

Rosie: You can’t like every girl that comes in here, you can’t just go off.

Adam: I don’t like every girl that comes in here, you asked us if I fancied Megan, no I don’t. Yeah, I probably do fancy Zara, but it didn’t really mean anything before you acted like a child.

Rosie: No, I haven’t acted like a child at all.

Adam: Well, we’ll agree to disagree.

Rosie: Oh, stop being a d***head, come on.

Adam: I’m not being a d***head.

Rosie: Like, it’s not nice when you come over and you sit like a metre away from me like you don’t want to touch me or don’t want to be near me.

Adam: Don’t. Oh my God you’re looking into everything. I’m just p****ed off at that, I think it is a bit ridiculous to be quite honest.

(Series 4 Episode 14, 2018: 11 min 55–13 min 42).

Considering gendered stereotypes that women are “crazy” and overly emotional (Sweet, 2019), when male contestants admit infidelity, they often use women’s emotionally charged reactions to justify their wrongdoings, thus shifting blame onto women. As such, during series five, Jordan used Anna’s emotionally charged reaction and “negativity” (Series 5 Episode 44, 2019: 44 min 42) to excuse why he sought a romantic connection elsewhere. In a similar regard, upon admitting his attraction towards Zara, Adam blamed Rosie for pushing him closer to Zara when explaining that while he was attracted to Zara, “it didn’t really mean anything before [Rosie] acted like a child” (Series 4 Episode 14, 2018: 13 min 22), thus highlighting how men blame their partners for their deceit.

Comparably, in series 5, Michael Griffiths justified his infidelity with Joanna Chimonides by blaming his former partner, Amber Gill. Michael and Amber were coupled during the first 2 weeks of *Love Island* but were isolated during a trial in which female islanders were sent to stay at separate villa, “Casa Amor,” where they were introduced to six new male contestants. While female islanders temporarily left for Casa Amor, male islanders welcomed six new female contestants in the main villa, with new arrivals intended to tempt islanders. Although Amber remained faithful to Michael during her stay in Casa Amor, Michael sought a romantic connection with a new arrival, Joanna. Unaware of this, Amber returned to the main villa to discover that Michael had chosen to re-couple with Joanna, and when asked why he chose to stray, Michael claimed he wasn’t himself during his relationship with Amber and without Amber in the villa, “the old me came back out” (Series 5 Episode 27, 2019: 4 min 27), prompting Amber to ask, “so that was my fault?” (Series 5 Episode 27, 2019: 4 min 32). Following this confrontation, Michael repeatedly accused Amber of being “childish” and “immature” which, feminist activist Scarlett Curtis suggested, serves as a form of manipulation (BBC Women’s Hour, 2019). As such, while Amber’s anger would be justified, she was not able to demonstrate this to Michael as an emotional response would confirm Michael’s accusations and substantiate the “crazy” woman stereotype.

Michael: I’ve haven’t been staying true to myself, biting my tongue in situations I wouldn’t usually bite my tongue in, overlooking things I wouldn’t usually overlook. I’ve been in that situation before and that’s why things haven’t worked out.

Caroline: [To Amber] How do you feel about what Michael just said?

Amber: A little bit confusing cause he could’ve said it to my face if he’s so straight up. Biting his tongue? Never heard that before, but what can I say, people just make s**t up on the spot don’t they?

Caroline: [To Michael] Why didn’t you say it to Amber before?

Michael: I have actually said it to Amber, but she said I made small situations into big situations and that’s why I actually can’t say nothing to her. I’ve stayed true to myself, and I’ve rised above it.

Caroline: [To Michael] But were you happy with Amber when she left for Casa Amor?

Michael: I was but at the same time I knew there was something wrong. I haven’t really been myself over the past 2 weeks and being here made me realise that I wasn’t myself. All the lads could see, and all the girls that came in, the old me that started came back out.

Amber: So that was my fault?

Michael: I couldn’t say nothing that I wanted to say to you because you thought I was making an issue and laughed whenever I said anything, because it’s always a massive joke to you.

Amber: It’s not a massive joke.

Michael: It seems that way, every time I say something to you, you either laugh in my face, which I think is really disrespectful, or you say I’m making a massive thing out of nothing.

Amber: What do you want us to do, cry? I’m not gonna say nothing right now.

(Series 5 Episode 27, 2019: 3 min 20–5 min 15)

### “The Money Shot”: Reinforcing Heteronormative Gender Roles Around Women’s Emotionality

In addition to the emotional manipulation enforced upon women from their male counterparts in *Love Island*, producers often deliberately manufacture scenes to evoke emotional responses from female islanders. Exploring the presentation of emotion in reality dating shows, Dubrofsky (2009) discusses how contestants’ emotions are often manipulated in order to capture the “money shot” (Grindstaff, 2002: 168), a term borrowed from film pornography to describe the orgasmic scene. In the context of television, Grindstaff (2002) and Dubrofsky (2009) suggest the “money shot” is utilized to depict the build-up of emotion, which erupts in a climactic, “spectacular and excessive” display (Dubrofsky, 2009: 355), whether this is the overpowering confession of love, or eruption of anger and jealousy. Accordingly, *Love Island* adopts this strategy, with producers manipulating contestants’ emotions to devise the “money shot.” However, as scenes of conflict and discomfort attract the highest viewing ratings, given how “conflict serves as a fundamental plot device in television” (Lauzen et al., 2006: 448) *Love Island* calls upon “audiences’ capacity for the unhappy ending” (Gray, 2009: 261) by manipulating contestants and creating conflict to evoke an emotional response. Particularly, with challenges centered around exposing deceit, such as the “Lie Detector Test” (Series 3 Episode 38, 2017), during which islanders are able to ask their partner questions while attached to a polygraph machine, and “License to Swill” (Series 3 Episode 21, 2017), where islanders hear an unfavourable quote about themselves and determine which fellow islander said this, conflict is encouraged in *Love Island* and expressions of anger often ensue.

However, in accordance with traditional gender roles, in which women adopt qualities of expressivity and emotionality (Hochschild, 1979), the “money shot” is consistently gendered towards women, thus affirming stereotypes surrounding women’s emotionality and irrationality. During series 4, *Love Island* viewers submitted over 2,500 complaints to the broadcasting watchdog, Ofcom, regarding the emotional manipulation of Dani Dyer during a challenge in Casa Amor (Shepherd, 2018). In this particular instance, Dani received an edited video clip of her partner, Jack Fincham, reuniting with his ex-girlfriend in separate villa, Casa Amor. In the clip, Jack appears to positively welcome his ex-partner, explaining to fellow islander Josh Ritchie, “oh my God, that’s the bird I was seeing before I came in here” (Series 4 Episode 24, 2018: 43 min 48). With existing insecurities, Dani interpreted Jack’s reaction as an indication of his intentions to reconnect with his ex-partner, thus becoming very distressed (Series 4 Episode 24, 2018: 43 min 54–45 min 20). However, the video was purposefully edited to cause Dani distress, as Jack remained faithful to Dani during his stay in Casa Amor and repeatedly expressed his feelings for her (Series 4 Episode 23, 2018: 12 min 00–12 min 12). Fundamentally, as “conflict serves as a plot device in television” (Lauzen et al., 2006: 448), producers purposely withheld images of reassurance to rather encourage an anxious response; thus exploiting Dani’s emotions to increase viewing figures and affirming gendered stereotypes on women’s paranoia and irrationality (Aslama and Pantti, 2006; Anderson and Ferris, 2016).

### “Do Bits Society”: Slut-Shaming and the Sexual Double Standard

Consistent with heteronormative ideals in intimate relationships, wherein roles are separated into masculine and feminine variables (Nahon and Lander, 2016), traditional “sexual scripts” (Gagnon and Simon, 1973) operate in *Love Island.* In accordance with the male sex drive discourse, in which it is expected that men “have stronger sexual urges and a greater need for sex than women” (Monaghan and Robertson, 2012: 142), *Love Island* reinforces gendered norms regarding physical intimacy by presenting men as occupying a dominant role in sex. Specifically, *Love Island* reinforces a sexual double standard in which men are celebrated for their sexual prowess, while women are shamed and punished.

As demonstrated by the “Do Bits Society” in series 4, where male contestants gathered daily to discuss sexual activities that had occurred the previous night, men achieve a sense of status and power through sex. Reminiscent of O’Neill’s research into the seduction industry (2018), in which it is argued that “the confirmation of a man’s sexuality through a woman is imbricated in his need to be validated as masculine by other men” (Buchbinder, 1998:110, cited in O’Neill, 2018: 59), male contestants achieve a sense of masculine status from their sexual relationships with women. However, the validation that men receive from sex is not self-validation, nor validation from their sexual partner, but rather, validation from fellow men (O’Neill, 2018). Accordingly, *Love Island* is a key example of the way in which “women provide heterosexual men with sexual validation, and men compete with each other for this” (Donaldson, 1993: 645, cited in Haywood, 2012: 73), as the “Do Bits Society” serves to provide male islanders with a sense of accomplishment and sexual conquest, thus establishing a hierarchy of masculinity among male contestants.

Comparatively, while men are rewarded with masculine status for their sexual accomplishments, women are shamed and punished. Accordingly, Zara Holland in series 2 was famously stripped of her Miss Great Britain title after engaging in sexual acts with Alex Bowen (Plunkett, 2016), while female contestants have since been repeatedly slut-shamed because of their “body counts,” with series 6’s Rebecca Gormley facing online trolling upon admitting having thirty sexual partners (Duffield, 2020). Fundamentally, the representation of female sexuality in *Love Island* perpetuates a simplistic and dichotomous vision of femininity, wherein one’s femininity is determined by their sexuality. Congruous to this narrative, the “good girl” is positioned against the “bad girl,” and while the “good girl” practices virtues of abstinence and chastity, the “bad girl” embraces her sexuality and occupies a dominant sexual role (Morris, 2017). Hence, consistent with how reality television shows present the “bad girl” as an “unruly woman,” characterized by her resistance to traditional gendered expectations of “ladylike behavior” (Gray, 2009: 270), Megan Barton-Hanson in series 4 and Maura Higgins in series 5 gained reputations as “man-eaters” (Series 5 Episode 15, 2019: 17 min 26) during their stay in *Love Island,* by virtue of their sex-positive attitudes and open discussions on sex. By representing sex-positive women in this manner, *Love Island*, therefore, perpetuates outdated assumptions of femininity, perceiving dominance in sex to be unfeminine and “unladylike” (Gray, 2009).

Moreover, *Love Island* draws attention to an unwritten expectation placed on women who openly discuss sex (McLaren, 2019), regarding the assumption that because a woman is sex-positive, she is sexually available and sexually experienced. Demonstrating this expectation in series 5, Maura Higgins confronted partner Tom Walker after he presented sexist behavior when stating to fellow male islanders “it’ll be interesting to see if she’s all mouth” (Series 5 Episode 18, 2019: 44 min 35), upon receiving news that Tom and Maura would be rewarded with an overnight stay in the villa’s private bedroom, the hideaway. Explaining to Tom that “me talking about sex doesn’t mean I’m going to jump on top of you” (Series 5 Episode 19, 2019: 17 min 19), Maura addressed the implicit assumption enforced upon sex-positive women and created a dialogue among islanders into how sex-positivity and openness around sex does not correlate with sexual promiscuity nor being “easy” (McLaren, 2019). Indeed, while men are often shown to present this belief in *Love Island,* this is an internalized assumption among both male and female islanders as, earlier in the series, Molly-Mae Hague was surprised to discover Maura had “only slept with five people” (Series 5 Episode 15, 2019: 17 min 07), given her “sensual” attitude (Series 5 Episode 15, 2019: 17 min 39) thus confirming assumptions into how sex-positive women are assumed to be sexually available and sexually experienced.

Tommy: Maura, on a scale of one to ten how much do you love sex?

Maura: Um, I’ve only slept with five people so.

Molly-Mae: What?

Lucy: Come off it.

Maura: I swear on my life.

Molly-Mae: You are messing!

Maura: Hello, I was in a 9-year relationship and a 2-year relationship. How would I have slept with any more?

Molly-Mae: Jesus Christ, really?

Maura: Jesus, everyone’s so shocked.

Amy: I thought you would’ve been a man-eater.

Molly-Mae: I thought you would’ve been in the f***ing numbers (gesturing a high amount) like the numbers.

Maura: Jesus, Molly!

Molly-Mae: Sorry, no disrespect but like cause obviously you’re so like, you know, sensual and you love it so I thought maybe the number would be higher than 5, but kudos to you. I’m proud of you, keep those numbers low.

Maura: Yeah, I don’t do one-night stands.

(Series 5 Episode 15, 2019: 17 min 01–17 min 52)

Further, by distancing herself from a narrative of sexual promiscuity when admitting “I wouldn’t just sleep with someone, I’ve never even had a one-night stand” (Series 5 Episode 19, 2019: 06 min 19), it can be argued Maura internalizes negative stigma surrounding women’s sexuality, in which women are criticized for having multiple sexual partners. As such, while sexual liberation is encouraged in contemporary society, restrictions remain to the extent that women having one-night stands and multiple sexual partners continues to be stigmatized.

## Conclusion

Upon analysis of the ten selected scenes, it can be argued that representations of gender in *Love Island* perpetuate sexist and heteronormative attitudes which serve to disadvantage women.

Specifically, this paper concedes that the use of the “money shot” (Grindstaff, 2002: 168) in *Love Island* reinforces a negative representation of women, in which women’s emotions are not only exploited to attract viewing figures (Aslama and Pantti, 2006), but to perpetuate stereotypes on women’s jealousy, paranoia, and irrationality. By manipulating female contestants’ emotions during production, as in the incident concerning Dani Dyer (Series 4 Episode 24, 2018), *Love Island* confirms traditional gender norms surrounding women’s emotionality. Moreover, with existing stereotypes surrounding women’s emotional instability, jealousy, and paranoia, men are able to call on women’s emotions and label them “crazy” when gaslighting them (Sweet, 2019). As demonstrated by Adam Collard accusing Rosie Williams of “looking into everything” (Series 4 Episode 14, 2018: 13 min 38) and Jordan Hames trivializing Anna Vakili’s suspicions, asking “am I not allowed to have a conversation with someone?” (Series 5 Episode 44, 2019: 43 min 59), men in *Love Island* often deny and trivialize their partners’ suspicions surrounding deception and infidelity by referring to stereotypes that posit women as emotional, paranoid and irrational, despite their doubts being justified. In a similar regard, recognizing that women tend to be more emotionally expressive, male contestants deliberately provoke their female partners to elicit an emotionally charged response. Adam Collard rolling his eyes and smirking during a confrontation with his partner Rosie Williams (Series 4 Episode 14, 2018) and Michael Griffiths blaming partner Amber Gill for his disloyalty (Series 5 Episode 27, 2019), therefore serve as examples of how men purposely manipulate women’s emotions and evoke an angry response to excuse their deception and confirm accusations that their partner is “crazy” (Sweet, 2019). Hence, by presenting female contestants to be overly emotional and irrational, outdated stereotypes surrounding women’s emotionality are reproduced in *Love Island*.

Moreover, *Love Island* reinforces gender norms surrounding the male sex drive discourse, in which an expectation remains that men “have stronger sexual urges and a greater need for sex than women” (Monaghan and Robertson, 2012: 142). Accordingly, when a female contestant embraces sex-positive attitudes and discusses sex openly, she is perceived as a “man-eater” (Series 5 Episode 15, 2019: 17 min 26) and negative assumptions are placed upon her. Maura Higgins in series 5 serves as an example of such assumptions, as fellow contestants perceived that because she holds sex-positive attitudes, she is sexually available and sexually experienced. Indeed, while Maura creates an important dialogue among islanders, regarding how sex-positivity does not correlate to sexual promiscuity (McLaren, 2019), this paper highlights the ongoing stigma surrounding women’s sexual promiscuity. As such, by Maura distancing herself from the “bad girl” stereotype when admitting “I’ve only slept with five people” (Series 5 Episode 15, 2019: 17 min 07) and explaining she “wouldn’t just sleep with someone, I’ve never even had a one-night stand” (Series 5 Episode 19, 2019: 06 min 19), it could be argued that Maura internalizes negative stigma surrounding women’s sexuality, in which women are criticized for having multiple sexual partners. Regarding this, while sexual liberation is encouraged in contemporary society, restrictions remain to the extent that women having one-night stands and multiple sexual partners continues to be stigmatized. *Love Island*, therefore, perpetuates outdated assumptions of femininity, wherein one’s femininity is determined by one’s sexual activity, thus perceiving dominance in sex to be unfeminine and “unladylike” (Gray, 2009). In this regard, *Love Island* draws attention to the existence of a sexual double standard in heterosexual relationships, whereby men are rewarded with masculine status for their sexual accomplishments, while women are shamed and punished.

Albeit examining a limited number of scenes from ITV2’s *Love Island*, this paper demonstrates the existence of traditional roles in contemporary intimate relationships and explores the extent to which such roles facilitate sexist attitudes towards women. Fundamentally, this paper depicts how, despite a cultural shift towards greater gender equality, traditional gendered ideals continue to exist in heterosexual relationships, which serve to disadvantage women. However, despite *Love Island* perpetuating outdated and sexist ideals, particularly regarding women’s emotionality and sexual promiscuity, exposure to these attitudes in a public forum allows the *Love Island* audience to occupy an objective stance, whereby viewers are able to critically assess intimate relationships and recognize problematic themes (BBC Women’s Hour, 2019), as indicated by the volume of Ofcom complaints received, and media coverage attracted by *Love Island.* Hence, while reality television may present problematic behaviors and attitudes towards women, such images provoke a public debate and encourage cultural change.

## Data Availability

The original contributions presented in the study are included in the article/Supplementary Material, further inquiries can be directed to the corresponding author.

## References

[B1] Anderson J. Ferris S. P. (2016). Gender Stereotyping and the *Jersey Shore*: A Content Analysis. KOME – Int. J. Pure Commun. Inq. 4 (1), 1–9. 10.17646/kome.2016.11

[B2] Aslama M. Pantti M. (2006). Talking Alone. Eur. J. Cult. Stud. 9 (2), 167–184. 10.1177/1367549406063162

[B3] Bauman Z. (2003). Liquid Love. Cambridge: Polity Press.

[B4] BBC Women’s Hour (2019). ‘Amika George, Teenage Campaigner Who Started #FreePeriods’ [Online]. Available through BBC Sounds (Accessed March 14, 2021).

[B38] Buchbinder D. (1998). Performance Anxieties: Re-producing Masculinity. St Leonards:Allen and Unwin.

[B5] Beck U. Beck-Gernsheim E. (1995). The Normal Chaos of Love. Oxford: Polity Press.

[B6] Cato M. Carpentier F. R. D. (2010). Conceptualizations of Female Empowerment and Enjoyment of Sexualized Characters in Reality Television. Mass Commun. Soc. 13 (3), 270–288. 10.1080/15205430903225589

[B39] Donaldson M. (1993). What is Hegemonic Masculinity? Theor. Soc. 22 (5), 643–645.

[B8] Dubrofsky R. E. (2009). Fallen Women in Reality TV. Feminist Media Stud. 9 (3), 353–368. 10.1080/14680770903068324

[B9] Duffield C. (2020). Love Island Fans Come to Rebecca's Defence after ‘slut-Shaming’ by Trolls. Yahoo! News [Online] (Accessed November 19, 2020).

[B40] Emmel N. (2013). Sampling and Choosing Cases in Qualitative Research London:Sage Publications Ltd.

[B10] Gagnon J. Simon W. (1973). Sexual Conduct: The Social Sources of Human Sexuality. London: Aldine Publishing.

[B11] Giddens A. (1992). The Transformation of Intimacy. Cambridge: Polity Press.

[B41] Glenn N. Marquadt E. (2013). Hooking Up, Hanging Out, and Hoping for Mr.Right New York:Institute For American Values.

[B42] Gough B. (2018). Contemporary Masculinities: Embodiment, Emotion and Wellbeing New York:Palgrave Macmillan.

[B12] Gray J. (2009). “Cinderella Burps: Gender, Performativity and the Dating Show,” in Reality TV: Remaking Television Culture. Editor Murray S. (New York: New York University Press), 260–277.

[B13] Grindstaff L. (2002). The Money Shot: Trash, Class, and the Making of TV Talk Shows. London: The University of Chicago Press. 10.7208/chicago/9780226309088.001.0001

[B43] Haywood C. (2018). Men, Masculinity and Contemporary Dating New York:Palgrave Macmillan.

[B14] Hochschild A. R. (1979). Emotion Work, Feeling Rules, and Social Structure. Am. J. Sociol. 85 (3), 551–575. 10.1086/227049

[B44] Illouz E. (2012). Why Love Hurts London:Polity Press.

[B45] Jamieson L. (1998). Intimacy London:Polity Press.

[B46] Kaplan D. Rosenmann A. Shuhendler S. (2017). What about Nontraditional Masculinities? Toward a Quantitative Model of Therapeutic New Masculinity Ideology. Men Masculinities 20 (4), 393–426.

[B47] Kempner J. (2014). Not Tonight: Migraine and the Politics of Gender and Health Chicago:University of Chicago Press.

[B15] Lauzen M. M. Dozier D. M. Cleveland E. (2006). Genre Matters: An Examination of Women Working behind the Scenes and On-Screen Portrayals in Reality and Scripted Prime-Time Programming. Sex Roles 55, 445–455. 10.1007/s11199-006-9100-5

[B16] Mason J. (2002). Qualitative Researching. London: Sage Publications. 10.4324/9780203471876

[B17] McGrath R. (2018). Love Island: Adam Collard’s Treatment of Rosie Williams Displays ‘Warning Signs’, Says Women’s Aid. Huffington Post [Online]. 10.1093/oso/9780198797852.003.0008 (Accessed February 25, 2021).

[B18] McLaren B. (2019). Tom and Maura’s Love Island Fight Is a Reminder that We Still Place Unfair Expectations on Women Who Are ‘sexually Open’. Grazia [Online] (Accessed March 25, 2021).

[B19] Monaghan L. F. Robertson S. (2012). Embodied Heterosexual Masculinities, Part 1: Confluent Intimacies, Emotions and Health. Sociol. Compass 6 (2), 134–150. 10.1111/j.1751-9020.2011.00447.x

[B48] Morgan S. Dennehy R. F (2004). 'Using stories to reframe the social construction of reality: A trio of activities. J. Manage. Educ. 28, 372–398.

[B20] Morris A. (2017). Good Girls VS Bad Girls: Exploring the Representations of Female Sexuality on ITV’s Love Island. The University of Birmingham [Online] (Accessed February 25, 2021).

[B49] Nahon D. Lander N. (2016). The Integrity Model: working with men, their intimacy issues, and their search for community. J. Men's Stud. 24 (24), 89.

[B21] O’Neill R. (2018). Seduction: Men, Masculinity and Mediated Intimacy. Cambridge: Polity Press.

[B50] Parsons T. (1959). The social structure of the family In: The Family, its Functions and Destiny editor Ashen, R.N. Harper:New York 241–274.

[B22] Plunkett J. (2016). Miss Great Britain Loses Title after Having Sex on ITV2’s Love Island. The Guardian [Online] (Accessed March 27, 2021).

[B23] Series 3 Episode 21 (2017). Love Island [Online]: ITV2, 21.00 28th June 2017. Available through ITV Hub (Accessed March 22, 2021).

[B24] Series 3 Episode 38 (2017). Love Island [Online]: ITV2, 21.00 18th July 2017. Available through ITV Hub (Accessed March 22, 2021).

[B25] Series 4 Episode 14 (2018). Love Island [Online]: ITV2, 21.00 19th June 2018. Available through ITV Hub (Accessed March 15, 2021).

[B26] Series 4 Episode 23 (2018). Love Island [Online]: ITV2, 21.00 29th June 2018. Available through ITV Hub (Accessed March 15, 2021).

[B27] Series 4 Episode 24 (2018). Love Island [Online]: ITV2, 21.00 1st July 2018. Available through ITV Hub (Accessed March 15, 2021).

[B28] Series 5 Episode 15 (2019). Love Island [Online]: ITV2, 21.00 19th June 2019. Available through ITV Hub (Accessed March 22, 2021).

[B29] Series 5 Episode 18 (2019). Love Island [Online]: ITV2, 21.00 23rd June 2019. Available through ITV Hub (Accessed March 15, 2021).

[B30] Series 5 Episode 19 (2019). Love Island [Online]: ITV2, 21.00 24th June 2019. Available through ITV Hub (Accessed March 15, 2021).

[B31] Series 5 Episode 27 (2019). Love Island [Online]: ITV2, 21.00 3rd July 2019. Available through ITV Hub (Accessed March 15, 2021).

[B32] Series 5 Episode 44 (2019). Love Island [Online]: ITV2, 21.00 23rd July 2019. Available through ITV Hub (Accessed March 15, 2021).

[B33] Shepherd J. (2018). Love Island: Ofcom Receive over 2,500 Complaints after ‘emotional Manipulation’ of Dani Dyer. Independent. [Online]. 10.1093/med/9780190466268.003.0015 (Accessed March 24, 2021).

[B34] Showalter E. (1981). “Victorian Women and Insanity,” in Madhouses, Mad-Doctors and Madmen: The Social History of Psychiatry in the Victorian Era. Editor Scull E. (Pennsylvania: University of Pennsylvania Press).

[B35] Sweet P. L. (2019). The Sociology of Gaslighting. Am. Sociol. Rev. 84 (5), 851–875. 10.1177/0003122419874843

[B36] York-Morris S. (2017). How to Recognize Gaslighting and Get Help. Healthline [Online] (Accessed March 22, 2021).

[B37] Zaccour S. (2018). Crazy Women and Hysterical Mothers: The Gendered Use of Mental-Health Labels in Custody Disputes. Can. J. Fam. L. 31 (1), 57–103.

